# Higher treatment effect after total knee arthroplasty is associated with higher patient satisfaction

**DOI:** 10.1007/s00167-020-06272-2

**Published:** 2020-09-12

**Authors:** Jörg Lützner, Franziska Beyer, Klaus-Peter Günther, Jörg Huber

**Affiliations:** 1 Department of Orthopaedic and Trauma Surgery, University Hospital Carl Gustav Carus, TU, Dresden, Germany; 2 Department of Orthopaedics, Stadtspital Triemli, Zurich, Switzerland

**Keywords:** Arthroplasty, replacement, knee, Patient-reported outcome measures, Patient satisfaction, Treatment outcome

## Abstract

**Purpose:**

The aim of this study was to investigate what influence the treatment effect after total knee arthroplasty (TKA) had on patient satisfaction.

**Methods:**

Prospective registry data of a University-based arthroplasty centre were used. 582 patients with unilateral bicondylar TKA were analyzed. Treatment effect (TE) was deduced from Oxford Knee Score (OKS) before and one year after surgery. Positive values correspond to improved symptoms (maximum 1.0 reflect no symptoms at all) and negative values correspond to deterioration of symptoms. Satisfaction on a visual-analogue scale from 0 to 10 and the willingness to undergo TKA surgery again was assessed one year after surgery.

**Results:**

The mean OKS improved from 22.1 before to 36.7 one year after TKA. Treatment effects ranged from 1.0 to –0.62 with a mean TE of 0.56. Taking an individual treatment effect of 0.2 as a cut-off between responder and non-responder, a total of 85.8% would be classified as responder after TKA. The mean satisfaction score with the TKA was 8.1. There was a significant correlation between the individual treatment effect and satisfaction after TKA (*p* < 0.001). The majority of patients (84.5%) would undergo surgery again. Patients not willing to undergo surgery again or those uncertain about this had lower satisfaction scores, a lower treatment effect and were more often female compared to patients who would undergo surgery again.

**Conclusion:**

Higher individual treatment effects resulted in higher patient satisfaction and willingness to undergo surgery again. However, some patients with a relatively low treatment effect were highly satisfied, which indicates the need for both information.

**Level of evidence:**

II.

## Introduction

The reported percentages of dissatisfaction after total knee arthroplasty (TKA) range from 14% up to 27% [5, 6, 23, 26, 28]. This is in contrast to excellent survival rates of 93% after 15 years in several arthroplasty registries [8]. It is, therefore, important to not only measure “objective” outcomes such as revision rates or range of motion (ROM), but also the individual outcomes experienced by the patients.

The classical concept of patient reported outcome measures (PROM) compares the mean scores before and after surgery for a cohort and gives the impression that all patients benefit more or less from the surgery. This is unfortunately not true and a small but relevant number of patients has unchanged or even worse symptoms after the surgery. These patients can easily be identified with the “treatment effect” method (TE). This method was introduced in total hip and knee arthroplasty [14] and demonstrated a good correlation to well established responder criteria [16]. This simple method can be applied to virtually any questionnaire, which assesses symptoms and impairments. It calculates the outcome for each patient individually.

Besides validated PROMs, the assessment of global satisfaction after knee replacement is recommended by several societies [30]. Different factors have been investigated which influence patient satisfaction. While it is still difficult to predict postoperative satisfaction before TKA [10], there are several well-acknowledged postoperative factors which contribute to patient satisfaction, including improvement in pain and function [28], absence of adverse events [7] and fulfilment of expectations [4, 5, 23]. However, not all studies have identified the same factors that influence patient satisfaction. Additionally, it has been suggested that regional differences may play a role in PROMs [27], which might explain these inconsistent findings.

The aim of this study was to investigate the influence of patient’s individual treatment effect on satisfaction after TKA in a large single-centre arthroplasty registry. We hypothesized that non-responders would be dissatisfied and that higher treatment effects would be associated with higher satisfaction scores.

## Materials and methods

For this study, the registry data of a University-based arthroplasty center, which started for TKA in January 2013, was used. All patients scheduled for TKA were asked to participate. Inclusion criteria for this study were patients with unilateral primary bicondylar TKA for primary or secondary knee OA and willingness to participate. Exclusion criteria included unicondylar knee arthroplasty (UKA), constraint TKA as well as TKA due to polyarthritis, neoplasia, a history of infection, no informed consent and patients who were not able to fill in the questionnaires reliably.

**Fig. 1:**
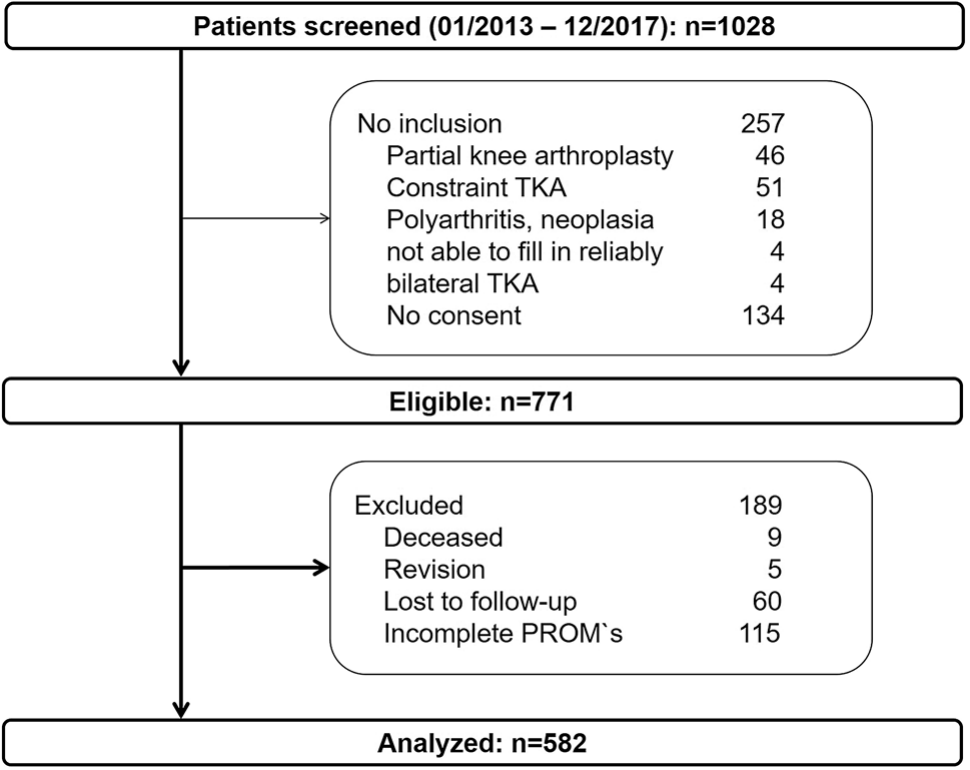
Study flowchart

From January 2013 to December 2017, a total of 1028 primary knee arthroplasties were performed. 257 patients did not meet inclusion criteria or had at least one exclusion criteria. From the eligible 771 patients, 189 were excluded within the one year follow-up period: 9 patients (1.0%) died not directly related to the surgery, 5 patients (0.5%) had revision surgery within the first year after surgery (4 patients due to infection, 1 patient due to periprosthetic femur fracture after a fall), 115 patients had incomplete follow-up data and 60 patients were lost to follow-up (Fig. 1). Patients analyzed were not different from the excluded patients regarding age, gender, BMI, comorbidities, indication for TKA and leg alignment (Table 1).

**Table 1:** Comparison of baseline characteristics of eligible patients given as mean (SD) and absolute (relative) frequencies

	Analyzed *n* = 582	Excluded *n* = 189	*p* value
Age at surgery (years)	68.7 (9.7)	70.1 (9.4)	ns
BMI (kg/m^2^)	30.9 (5.6)	31.4 (6)	ns
Gender			ns
Male	270 (46.4%)	79 (41.8%)	
Female	312 (53.6%)	110 (58.2%)	
Comorbidities			ns
ASA 1/2	304 (52.2%)	84 (44.4%)	
ASA 3/4	278 (47.8%)	105 (55.6%)	
Indication for TKA			ns
Primary OA	518 (89.0%)	172 (91.0%)	
Secondary OA	64 (11.0%)	17 (9.0%)	
Malalignment before surgery^a^	8.0 (4.2)	8.1 (4.7)	ns

^a^Deviation in degrees from a neutral leg axis

All patients received a cemented bicondylar TKA (Balansys, Mathys, Bettlach, Switzerland; Columbus or Vega, Aesculap, Tuttlingen, Germany; Nexgen, Zimmer Biomet, Warsaw, NJ) via a medial parapatellar approach without patellar resurfacing and completed a standardised postoperative rehabilitation program with pain-adapted full weight-bearing, initially with crutches.

The Oxford Knee Score (OKS) was used to assess symptoms and impairments before and one year after surgery. For each patient, the TE (treatment effect) was calculated TE = ([complaints before – after]/complaints before) using the OKS. For this calculation, the OKS had to be inversed, so that 0 equals no complaints and 48 maximal complaints. The TE for each patient was calculated as a numeric score: a positive number corresponds to improvement, zero represents no change and a negative number corresponds to deterioration. The TE`s were summarized in five “outcome categories”: excellent (TE > 0.95), good (TE > 0.50–95), moderate (TE > 0.2–0.5), unchanged (TE – 0.2 to 0.2) and worse (TE < − 0.2) [8]. Additionally, it has been demonstrated that a TE > 0.2 corresponds to validated responder criteria [16]. Therefore, patients with a TE > 0.2 have been considered as responders and patients with a TE ≤ 0.2 as non-responders.

At the one-year follow-up, patients were asked about their satisfaction with TKA surgery on a visual analogue scale from 0 (very dissatisfied) to 10.0 (very satisfied). Additionally, patients were asked if they would undergo TKA surgery again, if necessary. They were given five options to answer: definitely yes, likely, uncertain, unlikely, definitely no.

Patient characteristics, data from the surgery, comorbidities (ASA grade), and adverse events were recorded prospectively and together with the questionnaires assembled in the registry.

The study has been performed in compliance with the Helsinki Declaration and has been approved by the local ethics committee (EK135042014). All patients signed an informed consent.

### Statistical analysis

To detect a clinically relevant difference between responder and non-responder based on two grades on the satisfaction VAS with a standard deviation of 2.0, a power of 80% and *p* < 0.05 a minimum of 17 patients per group were necessary. With the actual group size of 74 non-responders, the power is 100%.

The data were analyzed using SPSS® software (release 26 for Windows®). Data was reported as means and standard deviation (SD) for continuous values and absolute and relative frequencies for categorical values, respectively. Univariate and multivariate regression analysis was performed to determine confounder, which had an independent influence on the satisfaction.

## Results

**Fig. 2:**
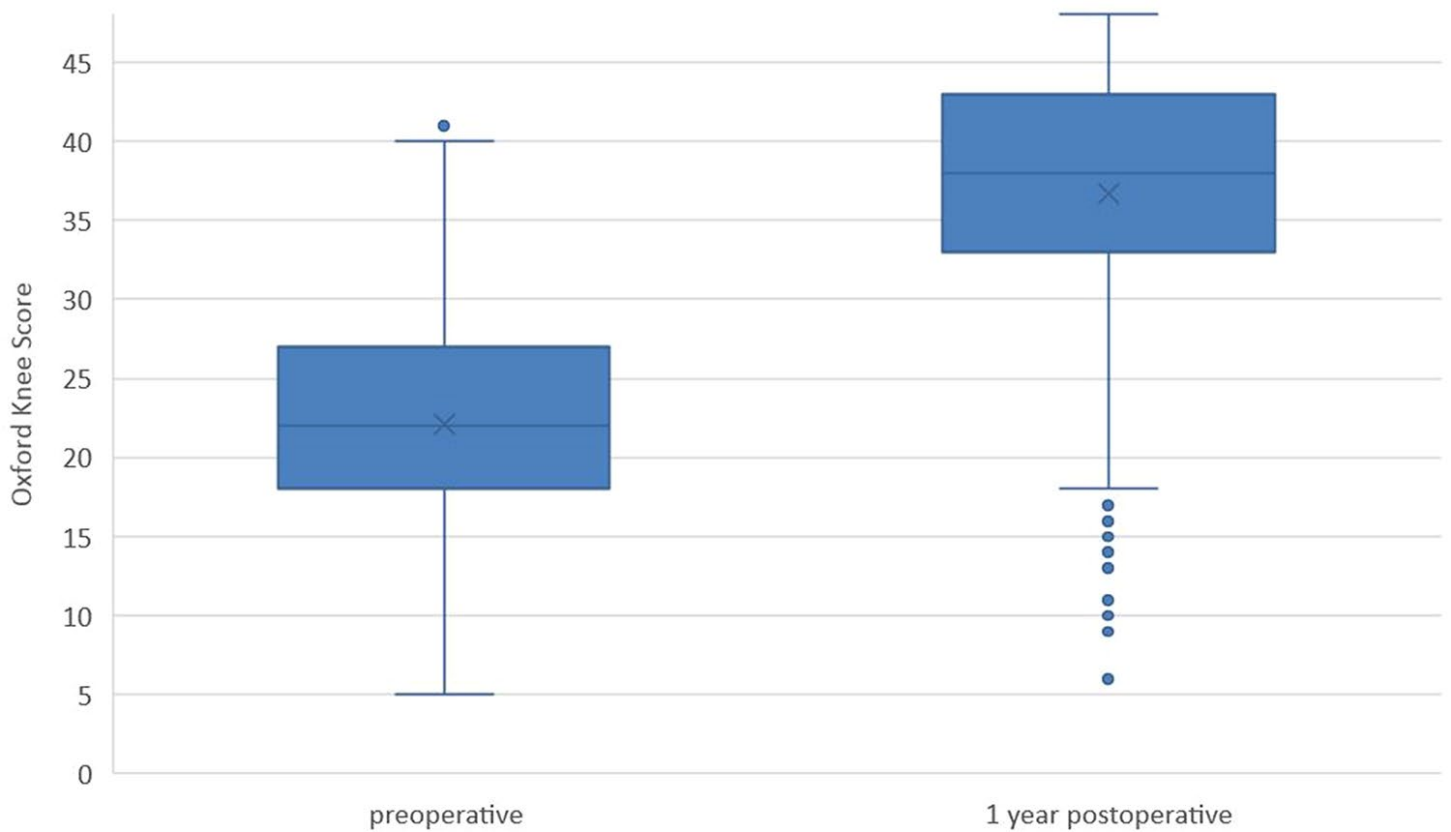
Box whisker plots for Oxford Knee Score before and one year after TKA

A total of 582 patients with TKA were included in this study. The mean OKS before TKA was 22.1 (SD 6.8) and one year after TKA 36.7 (SD 8.5) (Fig. 2) demonstrating a significant improvement.

**Fig. 3:**
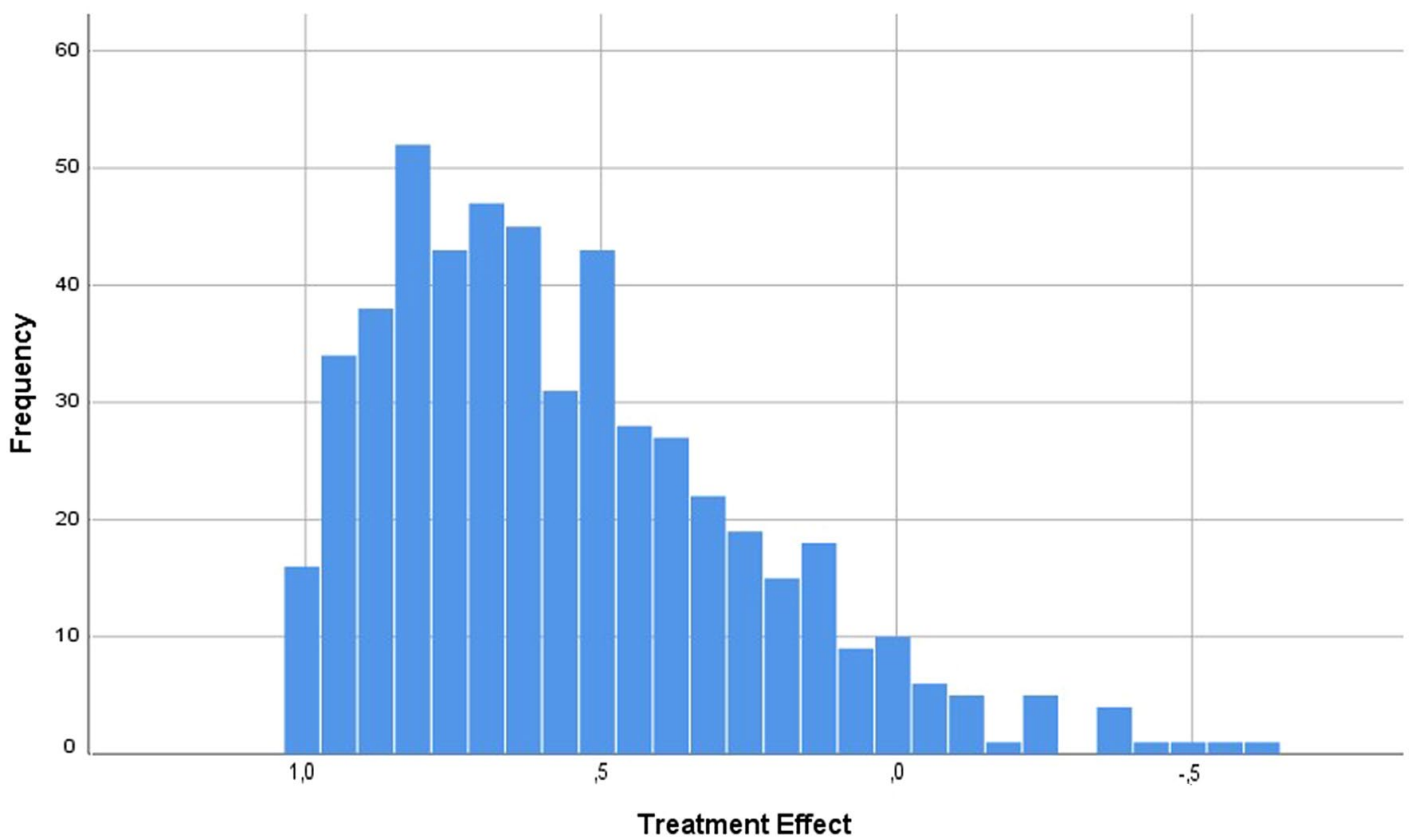
Individual treatment effects one year after TKA (excellent > 0.95, good > 0.5–0.95, moderate > 0.2–0.5, unchanged – 0.2 to 0.2, worse < 0.2)

Treatment effects ranged from 1.0 to –0.62 with a mean TE of 0.56 (Fig. 3). Taking an individual treatment effect of 0.2 as a cut-off between responders and non-responders, 448 patients (85.8%) were classified as responders after TKA and 74 patients (14.2%) as non-responders.

**Fig. 4:**
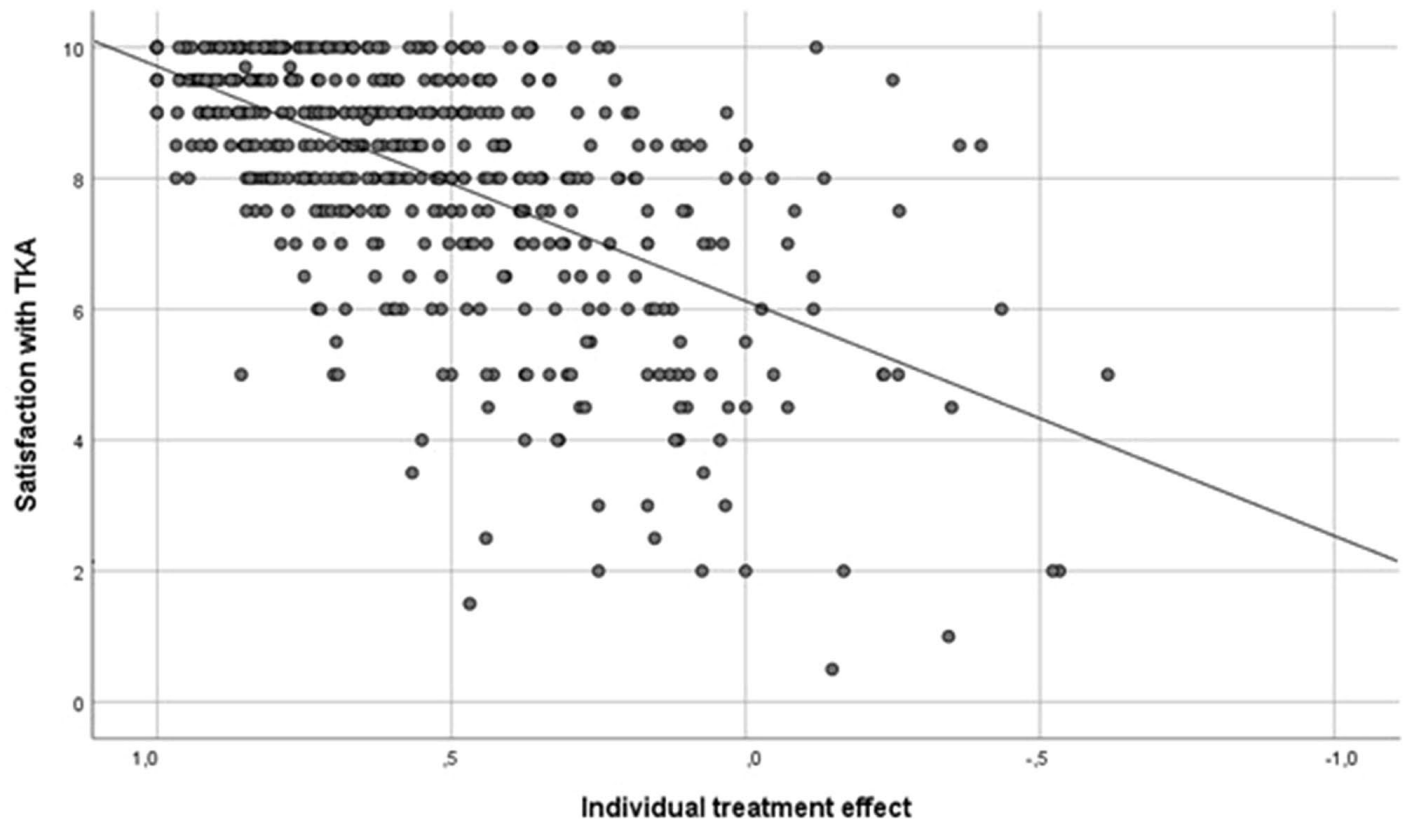
Correlation between satisfaction with TKA and individual treatment effect (excellent > 0.95, good > 0.5–0.95, moderate > 0.2–0.5, unchanged – 0.2 to 0.2, worse < 0.2)

Satisfaction score was available for 522 patients. The mean satisfaction score with TKA was 8.1 (SD 1.8) out of 10. Univariate analysis revealed that higher treatment effect, older patients, lower BMI and larger preoperative malalignment were associated with higher satisfaction scores. In the multivariate analysis, only the individual treatment effect remained a significant factor for patient satisfaction after TKA (regression coefficient 3.5, *p* < 0.001, Fig. 4). Surgical factors including implant brand and surgeon had no significant influence on satisfaction after TKA.

The satisfaction score was significantly higher with responders than non-responders [8.5 (SD 1.5) vs. 5.9 (SD 2.2), *p* < 0.001]. There were five patients, which had a very high satisfaction score of at least 9 but did not improve in the OKS with a TE of 0.2 or less. Four of these patients had severe problems with the contralateral knee and underwent surgery on the contralateral knee later on, one patient had a history of a stroke with residual symptoms.

The majority of the patients (84.5%) would undergo TKA surgery again, if necessary. However, one patient (0.2%) would definitely not undergo surgery again, 21 patients (4.0%) were unlikely and 59 patients (11.3%) uncertain to do so. Patients who would not undergo TKA surgery again or were uncertain about this had lower satisfaction scores, a lower treatment effect, were more often female and had lower malalignment before surgery than patients who would do so (Table 2). Responders would more frequently undergo surgery again. In a multivariate regression analysis, only satisfaction, treatment effect, responder and gender were confirmed to be independent variables, which influenced the willingness to repeat the TKA surgery.

**Table 2:** Factors influencing the willingness to undergo TKA surgery again given as mean (SD) and absolute (relative) frequencies

	Would you undergo the surgery again?		*p* value
	Yes, likely 441 (84.5%)	Uncertain, unlikely, no 81 (15.5%)	
Age at surgery (years)	68.3 (SD 9.6)	68.1 (SD 10.1)	ns
BMI (kg/m^2^)	30.7 (SD 5.6)	31.6 (SD 6.0)	ns
Gender			
Male	220 (49.9%)	27 (33.3%)	
Female	221 (50.1%)	54 (66.7%)	0.006
Indication for TKA			
Primary OA	392 (88.9%)	75 (92.6%)	
Secondary OA	49 (11.1%)	6 (7.4%)	ns
Leg alignment			
Before surgery^a^	8 (4.2)	6.1 (4.3)	0.002
Postoperatively^a^	2.1 (1.7)	2.5 (1.8)	ns
Correction	5.9 (4.2)	3.6 (4.8)	< 0.001
Treatment effect	0.61 (0.28)	0.26 (SD 0.32)	< 0.001
Responder	399 (90.5%)	49 (60.5%)	
Non-responder	42 (9.5%)	32 (39.5%)	< 0.001
Satisfaction with TKA	8.4 (SD 1.6)	6.4 (SD 2.2)	< 0.001
Oxford Knee Score [0–48 points]			
Before surgery	22.6 (SD 6.9)	21.0 (SD 6.2)	0.049
One-year follow-up	38.2 (SD 7.4)	28.4 (SD 9.0)	< 0.001
Improvement	15.6 (SD 8.2)	7.4 (SD 8.8)	< 0.001
OKS function subscale [0–100 points]			
Before surgery	52.2 (SD 16)	48.1 (SD 14.2)	0.031
One-year follow-up	74.7 (SD 16.2)	53.7 (SD 17.7)	< 0.001
Improvement	22.4 (SD 17.9)	5.6 (SD 17.2)	< 0.001
OKS pain subscale [0–100 points]			
Before surgery	43.3 (SD 15.9)	40.5 (SD 14.7)	ns
One-year follow-up	83.0 (SD 16.9)	63.0 (SD 21.5)	< 0.001
Improvement	39.7 (SD 19.8)	22.5 (SD 22.3)	< 0.001

^a^Deviation in degrees from a neutral leg axis

## Discussion

The most important finding of this study was the strong correlation between satisfaction after TKA and the individual treatment effect. However, some patients without any symptoms and complaints did not reach the highest satisfaction score and some patients were very satisfied but demonstrated only a low treatment effect. In patients with a high satisfaction score and a low treatment effect, relevant musculoskeletal and/or systemic comorbidities were existent. The negative effect of musculoskeletal comorbidities on outcome has already been demonstrated in hip arthroplasty [15].

Many studies have investigated variables, which influence satisfaction after TKA and tried to identify predictors. Several preoperative variables have been reported to influence satisfaction including body mass index (BMI) [9, 20], indication for TKA [7, 21], severity of osteoarthritis [19, 22, 29, 31], severity of symptoms [17, 20, 28] and mental health [1, 13, 28]. In this study, none of these variables had an influence on satisfaction. This is consistent with a large cohort study from the National Joint Registry for England and Wales [3], which found a low predictive capacity of preoperative variables. In a recent study, Goodman et al. [10] did also find no correlation between baseline measures and satisfaction two years after TKA in a large cohort.

Postoperative variables, that have been linked to patient satisfaction, include the absence of revision surgery [7], improvement of symptoms [3, 7, 10], improvement in walking distance and range of motion and fulfilment of expectations [4, 5, 23, 32].

In a systematic review, Gunaratne et al. [12] concluded that higher patient expectations, better function before surgery, less severe osteoarthritis, complications and less improvement in pain and function contribute to dissatisfaction after TKA. Baker et al. [3] found the perceived success of surgery, in terms of improved symptoms, had the largest influence on patient satisfaction. This is consistent with the present study in which the individual treatment effect (reduction of symptoms) had a significant effect on patient satisfaction and the willingness to repeat TKA surgery. The willingness of undergoing surgery again was taken as a surrogate for satisfaction with TKA. While patients who would undergo the surgery again probably perceived the TKA as a benefit, patients who would not do so or were uncertain probably had some kind of experience that made them doubt if TKA surgery was the right decision for their knee symptoms. Overall, patients with a higher treatment effect were more likely to repeat the surgery. Interestingly, 56.8% of patients classified as non-responders would undergo surgery again and probably perceived TKA as an improvement, even though these patients had only little or no improvement in symptoms, which could be measured with the OKS. Comorbidities, especially musculoskeletal comorbidities, might have been the reason for this. Symptoms in other major joints and/or systemic comorbidities reduce the ability for walking, stair climbing and other activities. Although the OKS questionnaire refers to knee issues, some patients might mix it up with other problems, which make the activity in question difficult. This might result in a low treatment effect, although the symptoms in the operated knee were improved.

Limitations of this study include a possible selection bias of patients at a university-based arthroplasty centre (high-risk patients). In this single-centre arthroplasty registry, the analyzed patients had a similar age but more serious comorbidities (47.8% ASA 3 and 4) than in the general German TKA population (33.7%) [18] and there were less female patients (53.6% vs. 61.2%). The revision rate of 0.5% within one year after TKA compares favourably with the revision rates reported in the German Arthroplasty Registry [11] (about 1.7% for unconstrained TKA), Australian Arthroplasty Registry [2] (about 1%) and National Joint Registry [24] (about 0.5%). The values of the Oxford Knee Score in our cohort before and one year after TKA corresponded well to published data from the NHS PROMs [25]. We, therefore, believe this cohort to be representative of TKA patients except for comorbidities. The final follow-up at one year after surgery does not exactly reflect the maximum improvement after TKA. It might, therefore, be possible that treatment effects and patient satisfaction are slightly worse than at a later follow-up. However, the majority of studies reporting patient satisfaction after TKA were based on a one-year follow-up. There was a relevant number of patients which could not be analyzed due to loss to follow-up or incomplete questionnaires. The baseline data of these patients were not significantly different from the analyzed patients. This might, therefore, have no relevant impact on the results.

Satisfaction after TKA was strongly correlated with the individual treatment effect, which represents the improvement of symptoms. However, satisfaction is a subjective rating and depends on other aspects of the treatment process too. Even patients with a relatively low treatment effect can be highly satisfied because they perceived the TKA surgery as successful. Therefore, both information—treatment effect and satisfaction—are necessary to finally judge the results of TKA surgery.

## Conclusion

Higher individual treatment effects resulted in higher patient satisfaction and willingness to undergo surgery again. However, some patients with a relatively low treatment effect were highly satisfied, which indicates the need for both information.
